# Effects of Node Fillet Radius on Compressive Properties and Energy Absorption of FDM-Fabricated PETG Auxetic Honeycombs

**DOI:** 10.3390/polym18182226

**Published:** 2026-09-12

**Authors:** Xin Kuang, Zhangyong Hu, Ruiyong Duan, Guangchao Zhan

**Affiliations:** School of Electromechanical and Intelligent Manufacturing, Huanggang Normal University, Huanggang 438000, China; 20144010@hgnu.edu.cn (X.K.); huzhangrun@163.com (Z.H.); duanruiyong@hgnu.edu.cn (R.D.)

**Keywords:** additive manufacturing, fused deposition modeling (FDM), PETG, auxetic honeycomb, node fillet, quasi-static compression, energy absorption, crushing stability

## Abstract

Node fillet radius has a pronounced effect on the compressive performance of fused deposition modeling (FDM)-printed polymer honeycombs, yet quantitative design guidelines for PETG auxetic structures remain scarce. The effects of gradient node fillet radii (R0–R7 mm) on the load-bearing capacity, deformation mechanisms and energy absorption of PETG auxetic honeycombs were investigated by quasi-static compression tests. Results reveal a dual competing mechanism: stress homogenization improves crushing stability, whereas nodal cross-section weakening reduces structural stiffness. Among the six configurations, R5 (35.7% radius-to-cell ratio) achieved the best balanced performance, with a peak strength of 0.35 MPa and specific energy absorption of 335.25 J/kg, and crushed progressively without catastrophic load drops. At R3 mm, nodal weakening raised the working stress and caused premature brittle fracture; at R7 mm, excessive filleting lowered peak strength to 0.30 MPa, slightly below the unfilleted control. Although unfilleted R0 showed the highest total energy absorption, specific energy absorption (342.62 J/kg) and efficiency (46.97%), its right-angle nodes induced disordered brittle collapse that compromises service safety. The resulting Pareto trade-off shows that moderate filleting best balances strength and energy absorption, providing parameter guidance for the topology optimization of 3D-printed polymer lightweight protective components.

## 1. Introduction

Polymer-based porous metamaterials have become a prominent research focus in additive manufacturing. By virtue of their high designability and tunable mechanical properties [1], they have been extensively adopted in lightweight functional applications such as aerospace protection, transportation impact mitigation, and smart wearables [2,3]. In particular, the integration of 4D printing technology and shape memory polymers further extends the application boundary of auxetic porous structures to reusable energy absorption scenarios [4]. Fused deposition modeling (FDM) is among the most widely used polymer 3D printing technologies. It enables rapid integrated fabrication and, combined with controllable costs and consistent dimensional accuracy, is particularly suitable for the large-scale production of lightweight polymer-based energy-absorbing components [5,6]. Auxetic honeycomb structures, commonly employed in engineering, allow precise regulation of the overall mechanical response and energy absorption characteristics by adjusting the nodal parameters [7]. This nodal regulation idea has also been verified in additively manufactured metallic structures, providing methodological reference for the topology optimization design in this work, thus meeting lightweight requirements in diverse scenarios. However, the stress concentration inherent in conventional right-angle nodes severely compromises the mechanical performance of FDM-printed parts and impairs the long-term service safety of the honeycomb structures [8].

The node configuration is a decisive factor governing the compressive response and failure mechanisms of polymer honeycomb structures. In conventional FDM-printed honeycombs, the widespread adoption of right-angle nodes readily induces stress concentration singularities under compressive loading, which accelerates crack initiation and rapid propagation, ultimately resulting in sudden brittle fracture and cliff-like load drops [8,9]. The crushing process consequently becomes disordered, substantially impairing the structural load-bearing stability and energy absorption efficiency and preventing the high toughness of PETG from being fully exploited [10]. Previous studies have demonstrated that topological modification through fillet radii offers an effective remedy for these shortcomings by optimizing the nodal stress distribution, reshaping the plastic deformation path, and enhancing the crashworthiness stability of the structure [11]. However, existing optimization efforts have predominantly focused on irregular bio-inspired topologies such as star-shaped, re-entrant, and chiral configurations [12,13], and have mainly employed metals and PLA as the base materials [14], while these studies provide fundamental theoretical support for the configuration optimization of porous structures, their findings cannot be directly transferred to the PETG auxetic honeycomb structures that are widely used in engineering.

Existing studies have developed various quantitative optimization frameworks for auxetic structural parameters, among which the Box–Behnken design based response surface methodology provides an efficient solution for multi-parameter and multi-objective performance tuning [15]. A review of the existing literature reveals that research on node modification for industrially common honeycomb structures remains insufficient, and readily implementable engineering design parameters are still relatively scarce [16,17,18]. Moreover, existing failure mechanism studies have predominantly been conducted on metals or brittle PLA materials, whose mechanical characteristics do not fully align with those of high-toughness PETG. Consequently, the gradient regulation laws governing the influence of fillet parameters on the compressive buckling, crack propagation, and interlayer failure of PETG honeycomb structures have yet to be systematically established. The optimal fillet parameter range for PETG honeycomb structures also remains unclear, and reliable experimental data to serve as a design basis are still lacking [5,19].

To address these research gaps, this study takes FDM-fabricated PETG auxetic honeycombs as the research object. Six groups of specimens with different node fillet radii were designed and subjected to quasi-static compression tests to quantitatively analyze the influence of fillet radius on load-bearing capacity, energy absorption, and crushing stability. Particular emphasis is placed on revealing the dual competing mechanism of nodal stress homogenization and load-bearing cross-section weakening, thereby providing experimental evidence for the topological parameter design of lightweight polymer energy-absorbing components.

## 2. Materials and Methods

### 2.1. Specimen Structural Design

**Figure 1:**
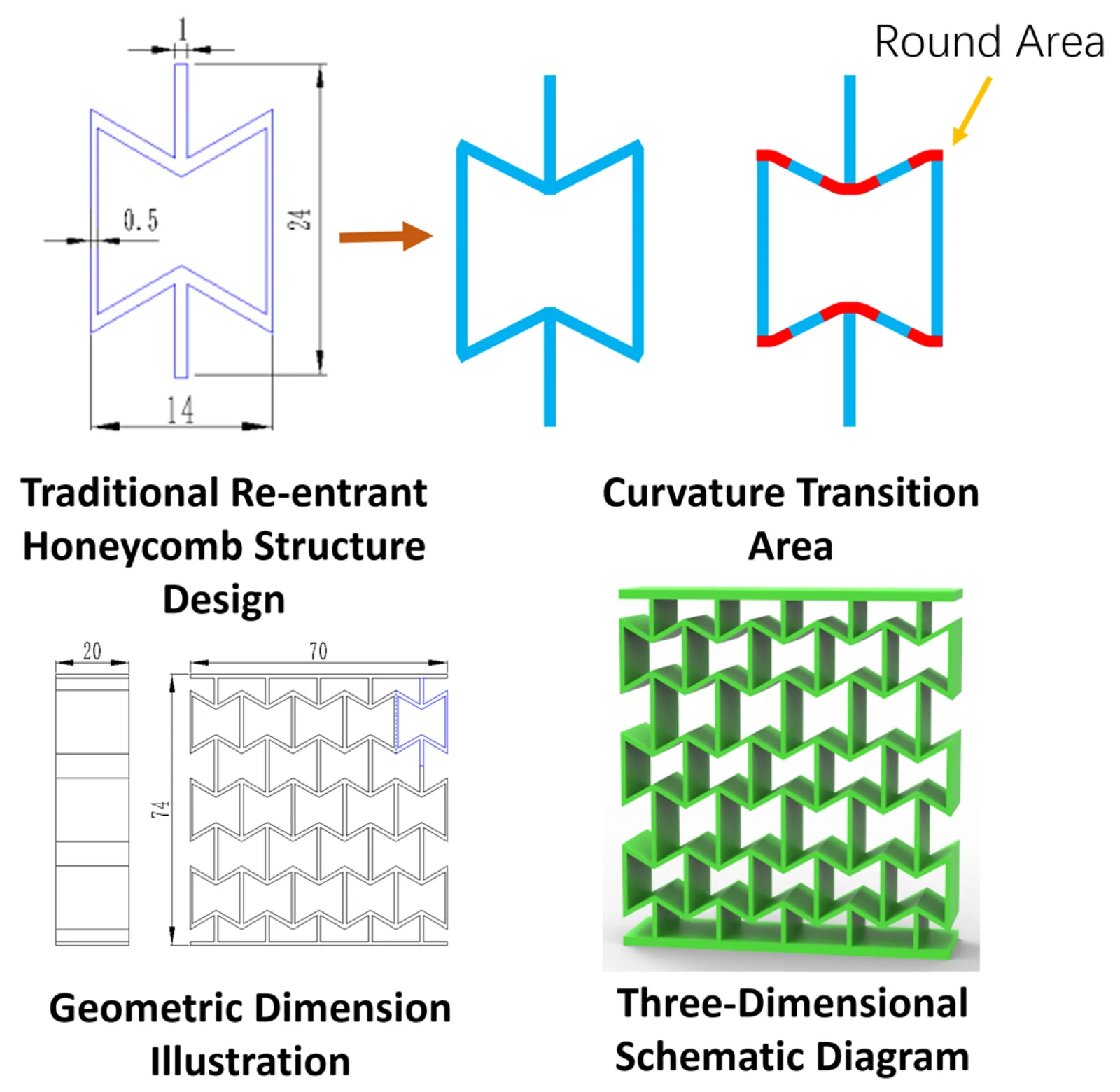
Schematic diagram of specimen geometry.

In this study, a re-entrant auxetic honeycomb structure, a common engineering configuration with negative Poisson’s ratio effect [14], was adopted. For terminological consistency, the structure is uniformly referred to as auxetic honeycomb throughout the manuscript. Following the principle of strictly controlling a single variable, all specimens were fabricated with uniform overall dimensions: height 74 mm, cross-section 70 mm × 20 mm, fixed cell spacing 14 mm, and rib width 1 mm, in order to eliminate, as far as possible, the interference of volume and porosity differences on mechanical properties and energy absorption [6]. The node fillet radius was taken as the sole variable. Based on the cell spacing of 14 mm, six groups of specimens with graded ratios were designed [11,12], the dimensionless ratios of fillet radius to cell spacing were set as follows: 0 (R0, control group without fillet), 10.7% (R1.5 mm), 21.4% (R3 mm), 35.7% (R5 mm), 46.4% (R6.5 mm), and 50.0% (R7 mm, corresponding to a nearly fully rounded node). All specimens exhibited regular cell topology and good structural symmetry, which reduces the influence of geometric anisotropy induced by topology on the compression test results [20]. It should be noted that geometric symmetry cannot completely eliminate the process anisotropy caused by FDM layer-wise fabrication and raster scanning direction. The unified printing orientation and process parameters for all specimens ensure the consistency of process anisotropy across groups, eliminating its interference with inter-group comparison. Quantitatively, as the fillet radius increases from R0 to R7 mm, the average specimen mass decreases from 24.04 g to 23.39 g, and the relative density decreases from 18.21% to 17.72%, with an overall variation amplitude of less than 2.8%. The measured mass and relative density of all specimen groups showed only minor fluctuations, verifying the comparability of the different fillet configurations (Figure 1).

### 2.2. Materials and 3D Printing Process

A commercial high-toughness PolyLite™ PETG filament (Polymaker, Suzhou, China) with a nominal diameter of 1.75 ± 0.02 mm was used. To eliminate the detrimental effect of moisture absorption on print quality and mechanical properties, all filaments were dried in a ventilated oven at 60 °C for 8 h prior to printing and then stored in a sealed desiccator until fabrication, thereby preventing bubble generation and interlayer interface defects during the printing process. According to the supplier’s technical data sheet and previously reported test results [10], this PETG grade exhibits a density of 1.27–1.28 g/cm^3^ and a thermal decomposition onset temperature as high as 410.5 °C, with negligible moisture volatilization in the low-temperature region, demonstrating excellent thermal stability.

**Figure 2:**
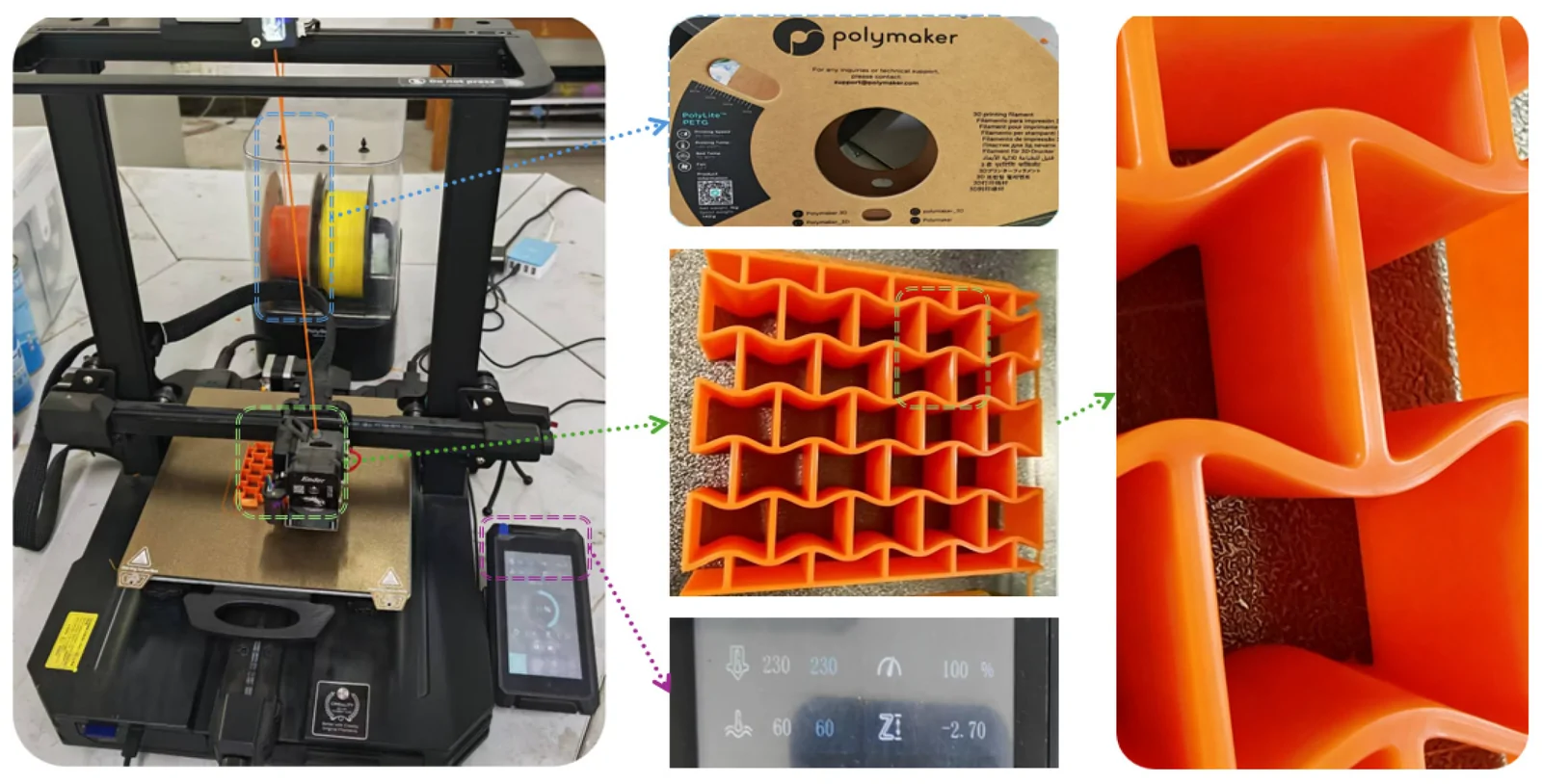
Photograph of the FDM-printed specimens.

All specimens were fabricated using an Ender-3 S1 Pro FDM printer (Creality, Shenzhen, China) equipped with a standard 0.4 mm brass nozzle. The standardized printing parameters were set as follows: nozzle temperature 230 °C, bed temperature 60 °C, layer height 0.20 mm, printing speed 50 mm/s, infill density 60%, cooling fan speed 100%, infill line width 0.4 mm, and retraction distance 4 mm. All specimens were printed with the in-plane honeycomb geometry laid flat on the build plate, and the 20 mm out-of-plane thickness direction was aligned with the printer Z-axis (layer stacking direction), which is perpendicular to the compressive loading direction. For the toolpath strategy, a single perimeter contour was printed first for each rib to ensure dimensional accuracy of ribs and nodes, followed by internal rectilinear raster infill. This raster infill scheme is a conventional toolpath configuration widely used in FDM thin-walled structure fabrication, and infill density and raster pattern have been verified as key process factors affecting the mechanical properties of printed parts [21]. The designed nominal rib wall thickness was uniformly 1 mm. The 60% infill density, set in Creality Slicer 4.8.2, refers to the infill ratio inside each rib cross-section, rather than the overall void fraction of the honeycomb structure. After printing, the specimens were left at ambient temperature for 24 h to allow full release of internal stresses, thereby eliminating the interference of residual stress on the mechanical tests. The uniform processing protocol ensured that no obvious macroscopic printing defects (such as stringing, insufficient extrusion, and macroscopic interlayer voids) were observed under visual inspection for all specimen groups. Microscopic defect characterization via microscopy or CT scanning will be carried out in future research to further verify the internal quality of specimens. Dimensional verification was conducted using a digital vernier caliper with 0.02 mm accuracy: the overall dimensional tolerance of all specimens was within ±0.15 mm, and the node fillet radius accuracy was within ±0.1 mm, which meets the standard manufacturing precision of FDM technology (Figure 2).

### 2.3. Quasi-Static Compression Test Protocol

**Figure 3:**
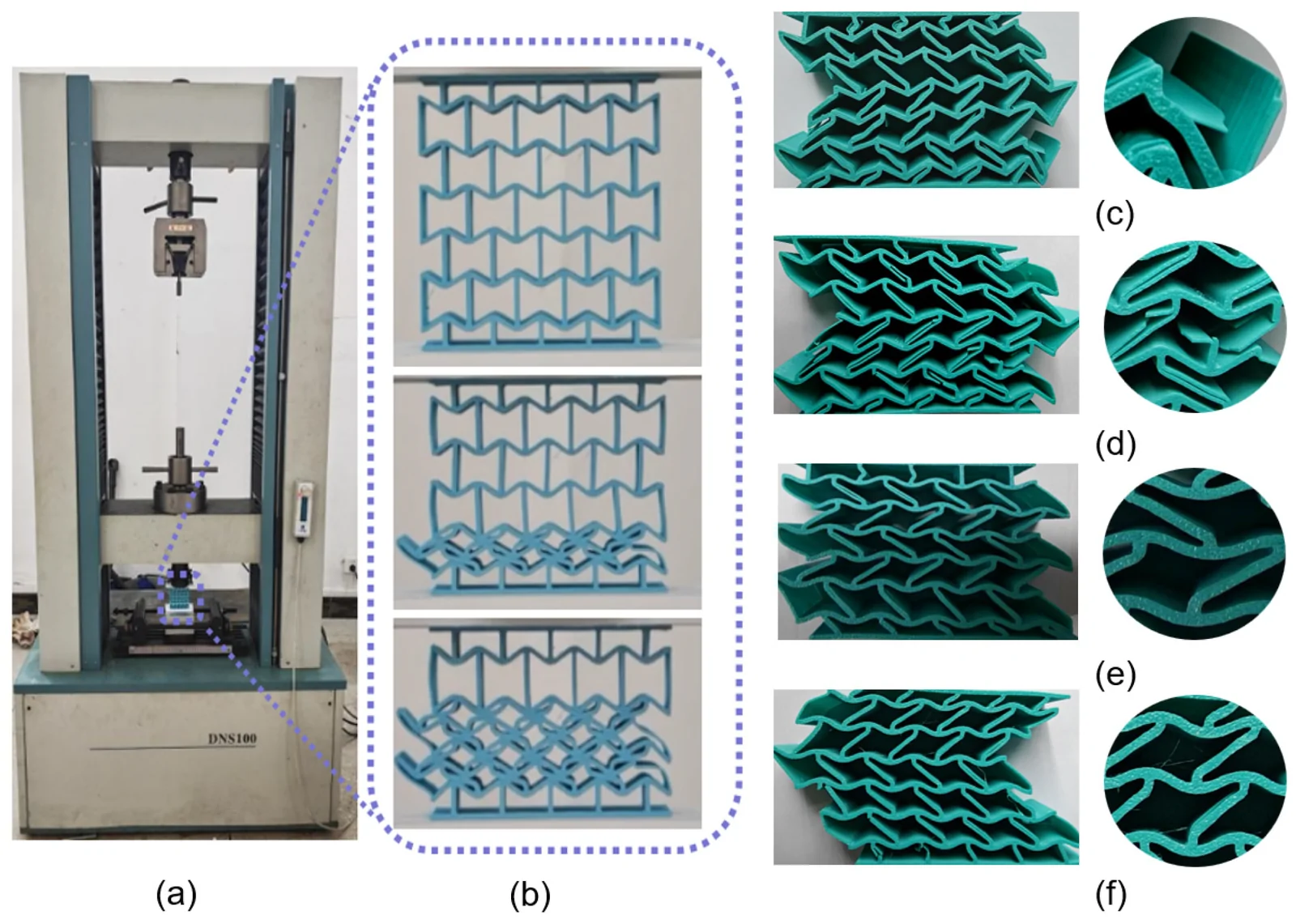
Quasi-static compression test setup, deformation process and typical failure morphologies of representative specimens. (**a**) DNS100 electronic universal testing machine; (**b**) Schematic of compression deformation process; (**c**) R0, brittle fracture at right-angle nodes; (**d**) R3, premature brittle fracture induced by nodal cross-section weakening; (**e**) R5, ordered progressive layer-by-layer plastic crushing; (**f**) R7, gentle compaction deformation. For each configuration: left = overall failure morphology, right = local enlargement of the nodal region.

The compression tests were conducted in accordance with GB/T 31930-2015/ISO 13314:2011 [22]. Although this standard was originally formulated for metallic porous materials, its core specifications—including displacement-controlled loading mode, plateau stage definition and energy absorption calculation framework—are universally applicable to cellular materials with different matrix materials. The loading principle and data processing rules specified in this standard are equally applicable to the quasi-static compression characterization of polymer-based porous structures [23] and have been widely adopted in recent studies on 3D-printed polymer honeycombs. A DNS100 electronic universal testing machine (Changchun Research Institute of Mechanical Science, Changchun, China) was used. All tests were performed under ambient temperature and pressure in a displacement-controlled mode at a constant loading rate of 5 mm/min, which satisfies the quasi-static loading condition and effectively avoids the influence of dynamic inertia effects on the test data. Taking into account the deformation characteristics of porous structures, the maximum compression displacement was set to approximately 40 mm (recorded up to 40.8 mm) to cover the entire regime from elastic deformation to plateau crushing, thereby enabling a comprehensive characterization of the principal mechanical responses and energy absorption properties of structures with different fillet configurations (Figure 3).

To ensure reliability and reproducibility, three parallel specimens were designed for each parameter set. Specimens with visible macroscopic printing defects (interlayer delamination, severe warpage, insufficient extrusion) were excluded before testing; the exclusion criterion was visually observable manufacturing defects rather than statistical outliers of mechanical test data. No numerical outlier detection algorithm was applied to the mechanical data, and all valid specimens were included in the data analysis. Supplementary specimens were fabricated and retested to guarantee valid sample size n = 3 for each group. All specimen groups exhibited satisfactory parallelism. The coefficients of variation for the key energy absorption indicators (total energy absorption and specific energy absorption) were below 5%, whereas that for peak strength ranged from 1.5% to 7.1% across the six groups (R0 3.0%, R1.5 7.1%, R3 1.7%, R5 1.5%, R6.5 2.9%, R7 5.5%). The wider spread of the peak-strength CV reflects the fact that the peak load is governed by local nodal defects, whereas the energy-absorption indicators are integrals over the entire crushing stroke and are comparatively insensitive to such local variations. Overall, the test repeatability was excellent, and the experimental data are reliable.

Because the assumptions of one-way ANOVA (normality and homogeneity of variances) cannot be meaningfully verified at n = 3 per group, and because post hoc multiple-comparison procedures would have very low power at this sample size, the group effect was instead assessed with the non-parametric Kruskal–Wallis test, whose null distribution was obtained by permuting the group labels (1,000,000 random permutations; complete enumeration of the 18!/(3!)^6^ ≈ 1.4 × 10^11^ possible assignments is computationally intractable). The null hypothesis of no difference among the six fillet configurations was rejected for every indicator examined: peak load and peak strength (H = 12.18, *p* = 0.0066), total energy absorption (H = 14.24, *p* = 0.0004), energy absorption efficiency (H = 14.52, *p* = 0.0002), load fluctuation coefficient (H = 13.21, *p* = 0.0020) and specific energy absorption (H = 14.73, *p* = 0.0001). These results indicate that the node fillet radius has a systematic effect on the measured mechanical response.

It should be emphasised what this test can and cannot establish. For a comparison between two groups of three specimens only C(6, 3) = 20 label assignments are possible, and complete separation of the two samples accounts for two of them, so the smallest attainable two-sided *p*-value is 0.100; no pairwise comparison can therefore reach *p* < 0.05, and no specific pair of configurations is claimed to differ significantly. For the principal comparison of interest, however, the R5 specimens exceeded the pooled remaining five configurations in peak load and peak strength (U = 42, two-sided *p* = 0.017, rank-biserial correlation r = 0.87), and all three R5 values exceeded all three R0 values (U = 0; one-sided *p* = 0.050, two-sided *p* = 0.100, i.e., at the resolution limit of this design). All *p*-values are two-sided unless stated otherwise. The non-monotonic trend is likewise supported: permutation tests on the Spearman correlation between peak load and fillet radius, computed at the level of individual specimens, give ρ = +0.713 (two-sided *p* = 0.013, n = 12) across the R0 → R5 interval and ρ = −0.949 (two-sided *p* = 0.0012, n = 9) across the R5 → R7 interval. Note that the R0 → R5 interval is not monotonic step by step—the R3 group mean (447.81 N) lies below that of R1.5 (469.12 N)—so the positive correlation characterizes the overall trend across the interval rather than a strictly increasing sequence.

The key geometric, material, FDM printing and quasi-static compression test parameters involved in this study are summarized in Table 1, which distinguishes fixed control parameters and variable nodal fillet radius.

### 2.4. Performance Evaluation Indicators and Calculation Methods

In accordance with the currently established evaluation framework for the crashworthiness and energy absorption of porous metamaterials [19], and based on the raw load–displacement data collected from the experiments, six key indicators were selected for quantitative calculation in this study. These indicators comprehensively assess the performance of each PETG specimen group from three perspectives: load-bearing capacity, energy absorption level, and crushing stability [17]. All indicators were calculated with unified dimensional standards and standardized unit conversions. The formulas are clearly defined with all parameters, and the calculation logic is closed-loop, ensuring that the data are scientifically sound and reproducible. The specific calculation formulas and parameter definitions for each indicator are presented below.

(1) Nominal Stress

Nominal stress is used to characterize the real-time load-bearing capacity per unit area of the structure, providing a direct reflection of the instantaneous stiffness level during compression. It is calculated as:

(1) $$\sigma =\frac{F}{S}$$

where *F* is the real-time compressive load in N, and *S* is the initial cross-sectional area of the specimen in mm^2^. In this study, the cross-sectional dimensions of the specimens were uniformly 70 mm × 20 mm, giving a fixed value of S = 1400 mm^2^. This nominal stress definition based on the initial gross cross-sectional area follows the standard specification for porous and cellular material compression tests (GB/T 31930-2015/ISO 13314:2011), which ensures consistent comparability of mechanical properties across different porous topologies. It should be noted that the effective load-bearing cross-sectional area of the ribs is itself part of the response under study and cannot be determined without arbitrary geometric assumptions; this normalization was therefore not performed. As the closest assumption-free proxy, the peak load normalized by the measured specimen mass (Fmax/m) is reported in Table S3: R5 ranks first under both normalizations, whereas the two lowest-ranked configurations, R0 and R7, interchange, so the core conclusion of this study is not affected.

(2) Nominal Strain

Nominal strain is employed to precisely characterize the degree of real-time compressive deformation during loading, eliminating the influence of specimen size differences on the deformation representation. It is calculated as:

(2) $$\varepsilon =\frac{\Delta L}{L_{0}}$$

where Δ*L* is the real-time compressive displacement of the specimen in mm, and *L*_0_ is the initial specimen height in mm. In this study, a fixed value of *L*_0_ = 74 mm was used.

(3) Total Energy Absorption

Total energy absorption represents the cumulative plastic deformation work of the structure from the elastic stage to the plateau stage. It is determined by integrating the load–displacement curve, with the integration performed over the full recorded range from the onset of loading to a common upper limit δd = 40.8 mm (the end of the recorded range), rather than to the individually determined densification onset of each configuration. A single common limit was adopted because the energy-efficiency criterion is not reached within the recorded range for several configurations (Section 3.3), so that no unambiguous per-group onset exists; the location of the efficiency maximum and the contribution of the terminal load-rise regime are reported there. This indicator therefore quantifies the energy dissipated over the entire deformation history covered by the test [24]. The calculation formula is:

(3) $$W=\int_{0}^{\delta_{d}}F(x)dx$$

where δd is the upper integration limit (in mm), taken as the common value of 40.8 mm for all configurations as explained above, and W is the total energy absorption (in J). In this study, trapezoidal integration was employed for the numerical calculation to ensure energy computation accuracy.

(4) Specific Energy Absorption

Specific energy absorption (*SEA*) is a core indicator for evaluating the energy absorption performance of lightweight porous structures. It eliminates the evaluation bias caused by differences in specimen mass, and characterizes the energy absorption capacity per unit mass of the structure. It is calculated as:

(4) $$SEA=\frac{W}{m}$$

where W is the total energy absorption of the specimen (in J) and m is the average mass of the three replicate specimens in that group (in kg; R0 = 24.04 g, R1.5 = 23.96 g, R3 = 23.78 g, R5 = 23.72 g, R6.5 = 23.51 g, R7 = 23.39 g). The unit of SEA is J/kg.

(5) Energy Absorption Efficiency

Energy absorption efficiency is used to characterize the degree of energy utilization of a structure under a given peak load, and it serves as a key indicator for evaluating the economic efficiency of energy absorption performance. Its value is jointly influenced by the peak stress magnitude and the smoothness of the crushing process [25]; it is calculated as follows:

(5) $$\eta =\frac{W}{\sigma_{\max}\cdot S\cdot \delta_{d}}\times 10^{-3}\times 100\%$$

where *σ_max_* is the peak stress of the specimen (in MPa), *S* is the initial (nominal) cross-sectional area (1400 mm^2^), and δd is the same common upper integration limit of 40.8 mm (in mm). The energy absorption efficiency is ultimately expressed as a percentage. A higher value of this indicator indicates that the structural crushing process approaches an ideal constant-force energy absorption state, reflecting better energy utilization economy.

(6) Load Fluctuation Coefficient

The load fluctuation coefficient is used to quantitatively characterize the deformation stability of the structure during the plateau crushing stage, serving as a key indicator for evaluating the engineering safety of energy-absorbing structures. A lower value indicates a more stable plateau load and a more controllable crushing failure mode. It is calculated as:

(6) $$CV=\frac{STDEV(F_{\mathrm{plateau}})}{AVG(F_{\mathrm{plateau}})}\times 100\%$$

where *STDEV*(*F*_plateau_) is the standard deviation of the load during the plateau crushing stage, and *AVG*(*F*_plateau_) is the mean load during the plateau crushing stage.

In this study, the plateau crushing interval was uniformly defined as a fixed displacement window of 2–30 mm. This window was verified to lie within the interval bounded by the elastic yield point (determined by the 0.2% offset method) and the densification initiation displacement (i.e., the peak of the energy efficiency–displacement curve) [26] for all 18 specimens, so that it covers the stable plateau stage while excluding the initial elastic rise and the terminal densification regime. The calculation rules for all performance indicators were kept consistent: the load fluctuation coefficient was calculated only from the load data within this 2–30 mm window, while the total energy absorption, specific energy absorption, and energy absorption efficiency were all integrated to the common upper limit δd = 40.8 mm, this approach avoids data errors caused by differences in interval selection and ensures the standardization and comparability of the experimental data.

## 3. Results and Analysis

### 3.1. Compressive Load–Displacement Curves and Deformation Modes

The compressive load–displacement curves of the six PETG specimen groups with different fillet radii all exhibit the typical three-stage deformation behavior of porous metamaterials, which is highly consistent with the compressive response characteristics reported for 3D-printed polymer porous structures [27]. In the initial loading stage, the load increases linearly with displacement, indicating a reversible elastic deformation state with stable stiffness and no plastic damage; the slope of the curve directly corresponds to the overall elastic stiffness of the structure. Upon entering the plateau crushing stage, the structure undergoes continuous plastic buckling and delamination crushing, with the load maintained in dynamic equilibrium; this interval constitutes the core stage for buffering and energy absorption. Once the pores are fully compacted and the ribs are squeezed into contact with each other, the load rises sharply with displacement, and the structure enters the densification stage, where the energy absorption capacity is essentially lost.

**Figure 4:**
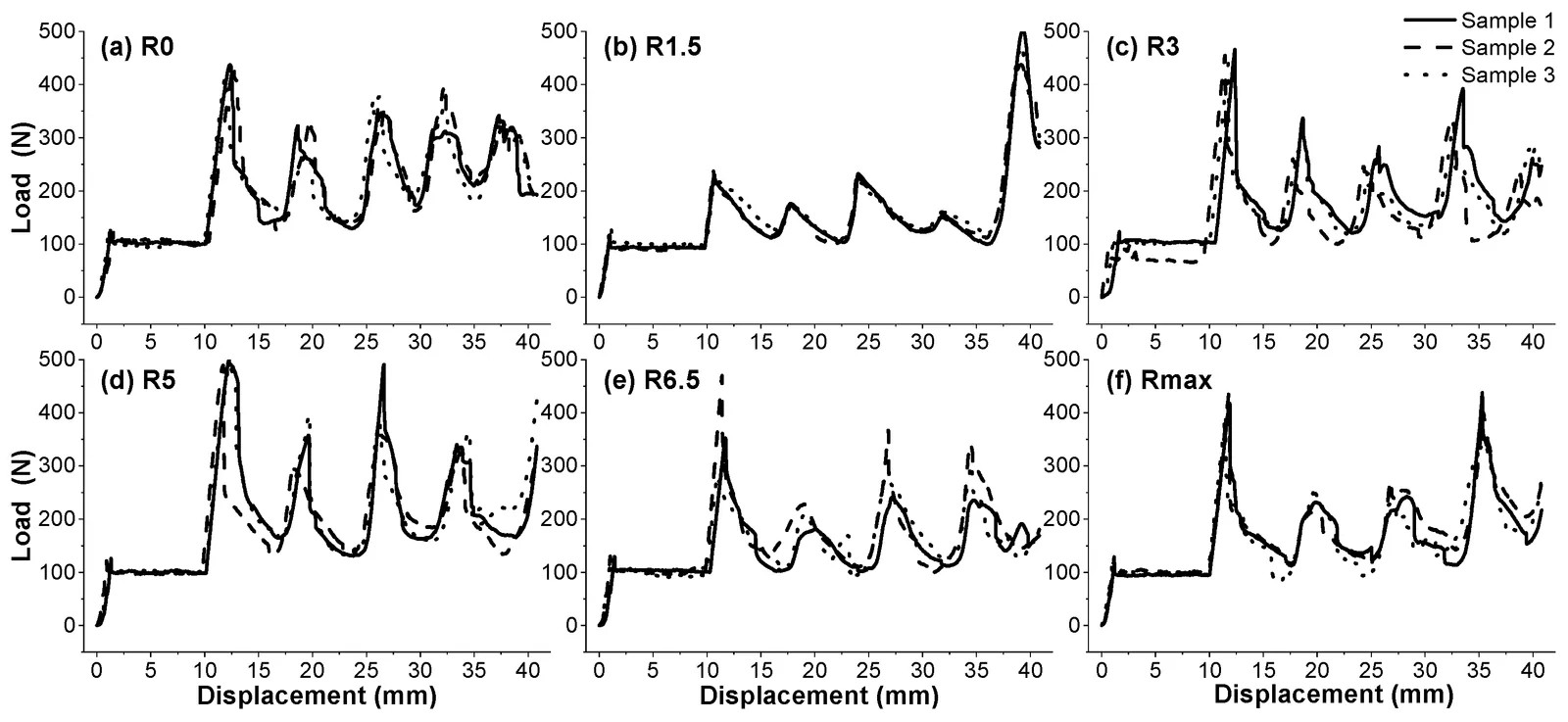
Compressive load–displacement curves of individual specimens in each group. (**a**) R0; (**b**) R1.5 mm; (**c**) R3 mm; (**d**) R5 mm; (**e**) R6.5 mm; (**f**) R7 mm.

The fillet radius exerts a pronounced gradient-regulating effect on the compressive response and deformation mode, with clear differences in the curve characteristics and failure behaviors among the specimen groups. As shown in Figure 4, the R0 specimens without a fillet exhibit prominent stress concentration at the right-angle nodes, and frequent cliff-like load drops occur in the middle and late stages of compression, corresponding to rapid crack propagation and sudden local fracture at the nodes (Figure 4c). This results in poor crushing continuity and disordered brittle failure, consistent with the failure behavior reported for porous structures with right-angle nodes [28].

For the R3 mm specimens, although the fillet eliminates the sharp-corner stress concentration singularity, the nodal load-bearing cross-section is more markedly weakened. The reduced cross-section leads to an elevated overall working stress level at the node, which superimposes with the residual stress gradient and presents brittle fracture behavior similar to stress concentration. The superimposed adverse effects lead to frequent load mutations with large drops during compression, making the brittle failure characteristics even more pronounced and preventing the attainment of the ideal progressive crushing energy absorption mode of porous structures (Figure 4d). In contrast, the R1.5 mm specimens exhibit a different behavior: the fillet partially mitigates the stress concentration at the nodes, and the stress concentration singularity at sharp corners is removed, leading to a smooth crushing process and minimal load fluctuation, with the lowest load fluctuation coefficient among all groups. The peak load and peak strength are also superior to those of the R0 and R3 specimens. Nevertheless, the circular-arc transition at the nodes alters the load-bearing fulcrum of the ribs, reducing the critical buckling load, which causes the compression process to enter the densification stage prematurely and thus shortens the effective energy absorption interval.

The R5 specimens with an intermediate fillet radius exhibited smooth and regular curves without pronounced load drops. The plateau-stage load exhibited ordered progressive fluctuation, and the structure displayed a typical layer-by-layer progressive plastic crushing mode with an orderly and controllable deformation process (Figure 4e). For the large-fillet specimens R6.5 and R7, the nodal stress concentration was also completely eliminated, and the crushing process was even more stable. However, excessive rounding of the nodes substantially reduced the effective load-bearing cross-section, leading to a marked decrease in the overall structural stiffness and a lower load baseline in the plateau stage, which in turn limited both the load-bearing capacity and the upper bound of energy absorption (Figure 4f). These deformation patterns are highly consistent with the common conclusion summarized in reference [6], which states that structural configuration optimization can reshape the plastic deformation path and balance crushing stability with load-bearing performance.

**Figure 5:**
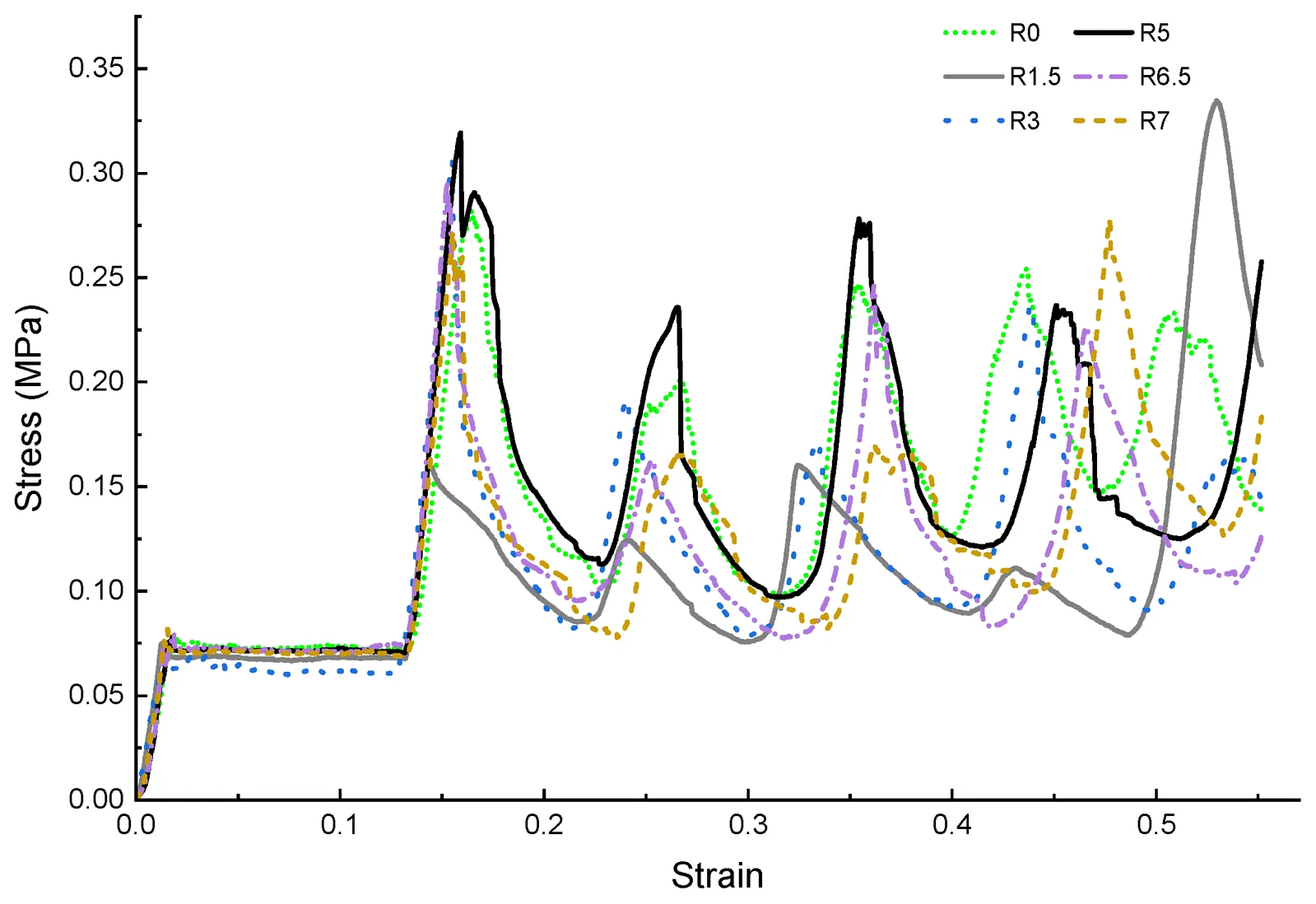
Compressive stress–strain curves of auxetic honeycomb structures with different node fillet radii.

As shown in Figure 4 and Figure 5, the compressive deformation of all six specimen groups followed the typical three-stage evolution of porous structures, and the differences in fillet radius were directly reflected in the smoothness of the plateau stage and the overall load level. The R0 and R3 specimens exhibited severe plateau-stage fluctuations with frequent load mutations and a highly disordered crushing process. In contrast, the R5 specimens maintained a relatively high load baseline while displaying a comparatively stable crushing process. The R1.5, R6.5, and R7 specimens showed smooth overall crushing behavior but with a reduced load-bearing stiffness. This finding, namely that topological configuration optimization can balance crushing stability with load-bearing performance, is consistent with previous studies [29].

### 3.2. Peak Load and Peak Strength Analysis

**Figure 6:**
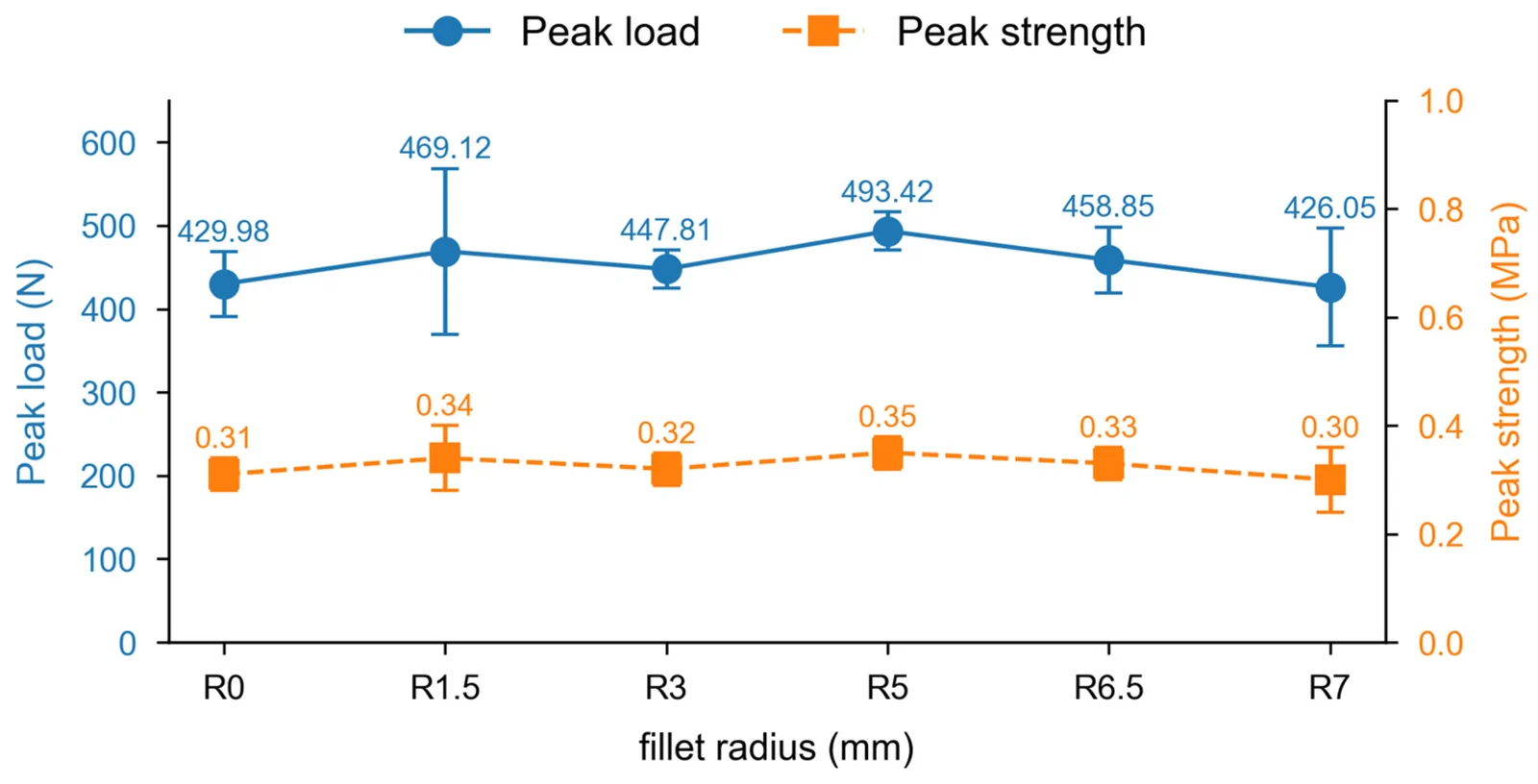
Comparison of peak load and peak strength.

Table 2 and Figure 6 present the statistical results of peak load and peak strength for each specimen group. A clear nonlinear gradient relationship between fillet radius and load-bearing performance can be observed. Taking the unfilleted R0 specimens as the baseline, their peak load and peak strength were 429.98 N and 0.31 MPa, respectively. The R0 specimens were susceptible to early local failure induced by stress concentration, and their load-bearing performance was inferior to that of the R3 and R5 specimens.

For the small-fillet groups, the R1.5 mm specimens exhibited a peak load of 469.12 N and a peak strength of 0.34 MPa, both higher than those of the R3 and R0 specimens. This confirms that an appropriate small fillet can enhance the peak load-bearing capacity by alleviating nodal stress concentration [11]. The R3 specimens exhibited a peak load of 447.81 N and a peak strength of 0.32 MPa, which were slightly higher than those of R0 but still lower than those of R1.5.

The R5 mm specimens achieved the highest peak load of 493.42 N and peak strength of 0.35 MPa, corresponding to an improvement of 14.75% in peak load and 14.75% in peak strength, relative to R0. These specimens possessed sufficient stiffness in the initial elastic stage and outstanding ultimate load-bearing capacity, achieving an optimal balance between nodal stress optimization and retention of the effective load-bearing cross-section.

When the fillet radius was further increased from R5 mm to R7 mm, the effective load-bearing cross-section at the nodes continued to shrink, leading to a progressive reduction in overall structural stiffness and a graded decline in peak load and peak strength. Notably, the peak strength of the R7 mm specimens was only 0.30 MPa, even lower than that of the unfilleted R0 specimens (0.31 MPa). This trend is consistent with the general rule that the nodal cross-sectional size of porous structures is positively correlated with mechanical load-bearing performance [30]. From the perspective of energy absorption, although the R3 mm fillet slightly improved the peak load-bearing capacity, it failed to enhance crushing stability and instead shortened the effective energy absorption interval, resulting in reduced total energy absorption and specific energy absorption.

As shown in Table 2 and Figure 6, the R5 mm intermediate fillet scheme maintained a high ultimate load-bearing capacity while suppressing brittle failure, demonstrating the best overall load-bearing performance among all groups. Detailed energy-absorption comparison is presented in Section 3.3.

### 3.3. Energy Absorption Performance Analysis

**Figure 7:**
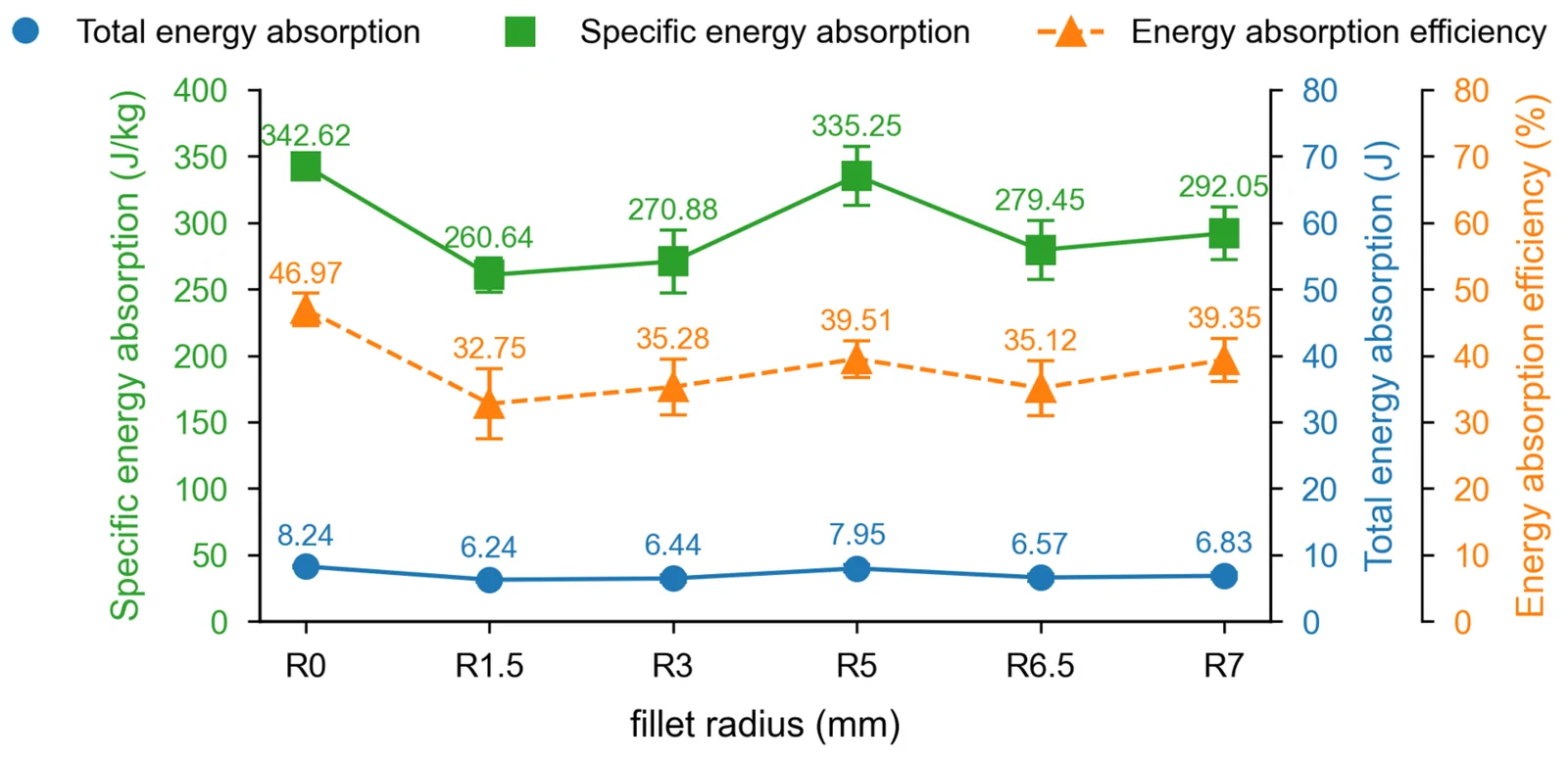
Comparison of energy absorption performance.

Total energy absorption, specific energy absorption, and energy absorption efficiency are the core indicators for evaluating the energy absorption performance of porous structures [31]. The energy absorption parameters of each specimen group are listed in Table 3 and Figure 7.

Densification displacement. The densification onset was located by the energy-efficiency method from the maximum of η(x) = W(x)/(Fmax·x). The efficiency maximum ranged from 35.1 to 40.8 mm across the 18 specimens. It fell strictly inside the recorded range for seven of the 18 specimens—R0 (39.2 and 40.2 mm for two of the three replicates), R5 (36.0 and 35.1 mm for two of the three replicates) and R6.5 (36.8, 38.2 and 38.2 mm)—whereas for every replicate of R1.5, R3 and R7, for one R0 replicate and for one R5 replicate, η(x) was still increasing at the end of the record (40.8 mm), so that the energy-efficiency criterion was not reached within the recorded range and the true onset lies beyond it. Because the per-group onset cannot therefore be located on a common and unambiguous basis, a uniform upper integration limit of δd = 40.8 mm (≈55% nominal strain, the end of the recorded range) was adopted for all groups; W, SEA and η were integrated to this common limit (Table 3). This limit necessarily includes the terminal regime in which the load rises again, and the size of that contribution differs among the configurations: the mean load over the final 5 mm (35.8–40.8 mm) is 2.1 (R1.5), 1.5 (R0), 1.4 (R7), 1.2 (R3), 1.1 (R6.5) and 1.1 (R5) times the mean plateau load over 2–30 mm, and the corresponding share of W is 23.4, 16.4, 16.3, 13.8, 13.1 and 13.0%, respectively. The terminal rise is thus most pronounced for R1.5, whose low plateau load makes the relative increment largest, and least pronounced for R5 and R6.5; because the same limit is applied to all groups, this affects the absolute magnitude of W but not the internal consistency of the comparison.

The ranking of total energy absorption is R0 > R5 > R7 > R6.5 > R3 > R1.5. The unfilleted R0 specimens, which did not suffer cross-sectional loss from node cutting, exhibited the highest total energy absorption and energy absorption efficiency. However, this high efficiency was obtained under a relatively low peak stress, and the intermittent brittle collapse induced by the right-angle nodes disrupted the crushing continuity, dissipating a large amount of plastic work in crack propagation and resulting in insufficient practical buffering reliability. The R5 mm specimens with an intermediate fillet radius showed total and specific energy absorption only slightly lower than those of R0. Owing to the orderly progressive crushing mode that suppressed ineffective energy dissipation, the R5 specimens achieved a well-balanced energy absorption efficiency and the best comprehensive energy absorption performance. For the R3 mm specimens, although the sharp-corner stress singularity is removed, the pronounced nodal cross-section weakening elevates the local working stress and induces brittle fracture at an early stage of compression, which greatly shortened the effective energy absorption interval and led to relatively low total energy absorption, specific energy absorption, and energy absorption efficiency. The R1.5 mm specimens exhibited a smooth crushing process but a low average plateau stress, resulting in limited energy storage per unit deformation and the lowest energy absorption efficiency among all groups. The large-fillet R6.5 and R7 mm specimens showed gentle crushing processes and moderate energy absorption efficiency, but the excessive weakening of the nodal cross-section reduced the overall stiffness, making them unsuitable for high-level energy absorption scenarios.

These performance differences are consistent with the conclusion of previous studies that an inherent trade-off exists between crushing stability and load-bearing capacity [32].

### 3.4. Crushing Stability and Comprehensive Performance Evaluation

**Figure 8:**
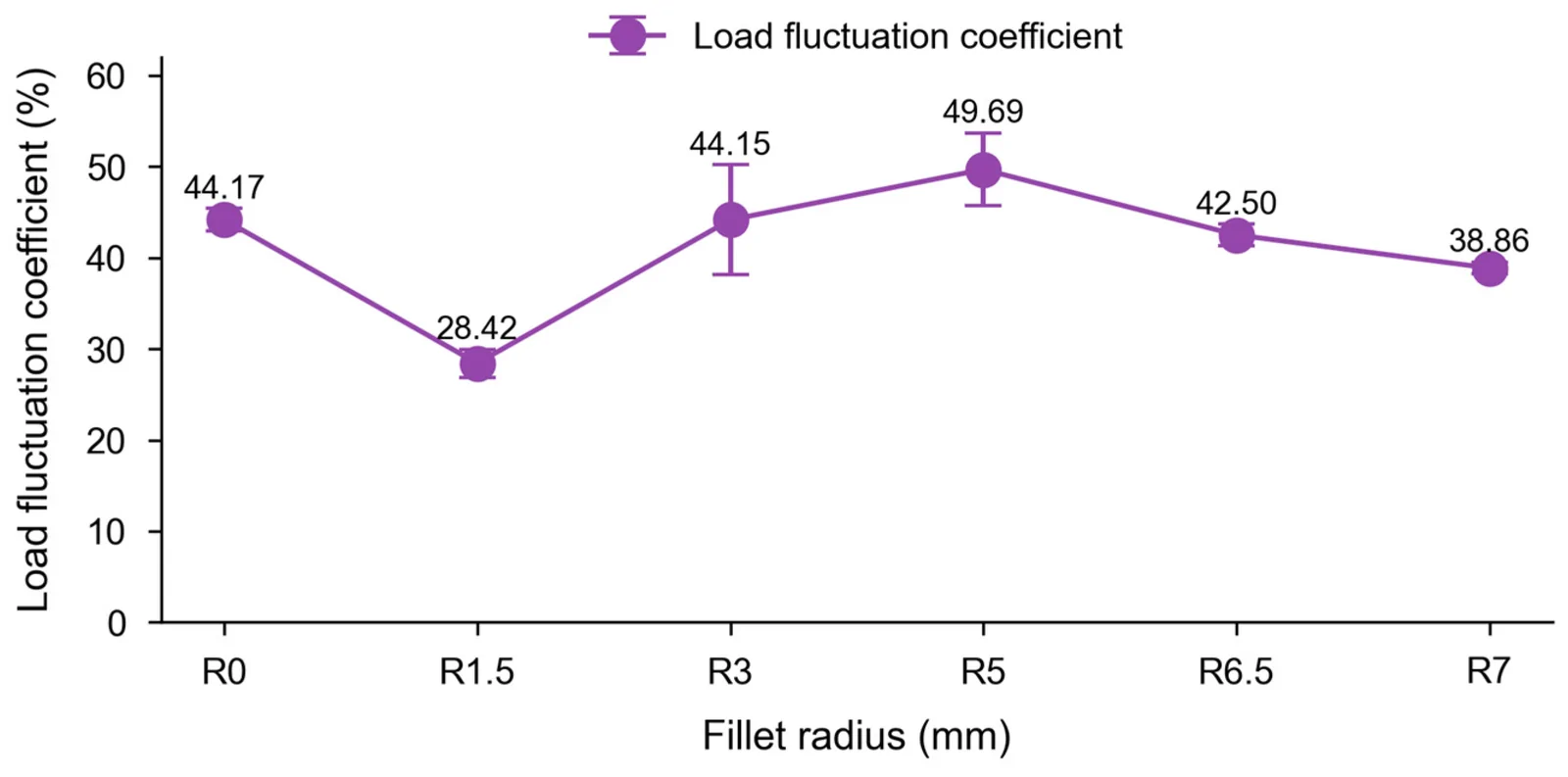
Comparison of load fluctuation coefficient.

The load fluctuation coefficient (CV) is an auxiliary quantitative indicator that reflects the overall dispersion degree of plateau-stage load, and cannot be used as the primary criterion for crushing stability. From the perspective of engineering safety, the core of crushing stability is the absence of catastrophic sudden failure and controllable deformation process. CV alone cannot distinguish between gradual oscillation from ordered progressive buckling (controllable and non-catastrophic) and abrupt drop from brittle fracture (high-risk and catastrophic). Therefore, crushing stability must be evaluated primarily by failure mode, supplemented by the CV value. The statistical results of the load fluctuation coefficient for each specimen group are presented in Table 4 and Figure 8.

As shown in Table 4, the R1.5 mm specimens exhibited the lowest load fluctuation coefficient (28.42%) among all groups, indicating the smallest fluctuation in crushing load. The R5 mm specimens showed the highest coefficient (49.69%); however, this fluctuation originated from layer-by-layer ordered plastic buckling rather than sudden load drops. The R3 mm specimens exhibited a coefficient of 44.15%, but their fluctuation was accompanied by brittle instantaneous fracture. Therefore, although the CV value of R3 was slightly lower than that of R5, the R3 specimens exhibited the worst crushing stability in terms of failure mode. It should be emphasized that the load fluctuation of R5 originated from ordered progressive plastic buckling without catastrophic load drops associated with brittle fracture, whereas the fluctuations of R0 and R3 were associated with brittle instantaneous fracture and thus pose higher service risks. The CV value alone cannot distinguish these two fundamentally different failure modes.

**Figure 9:**
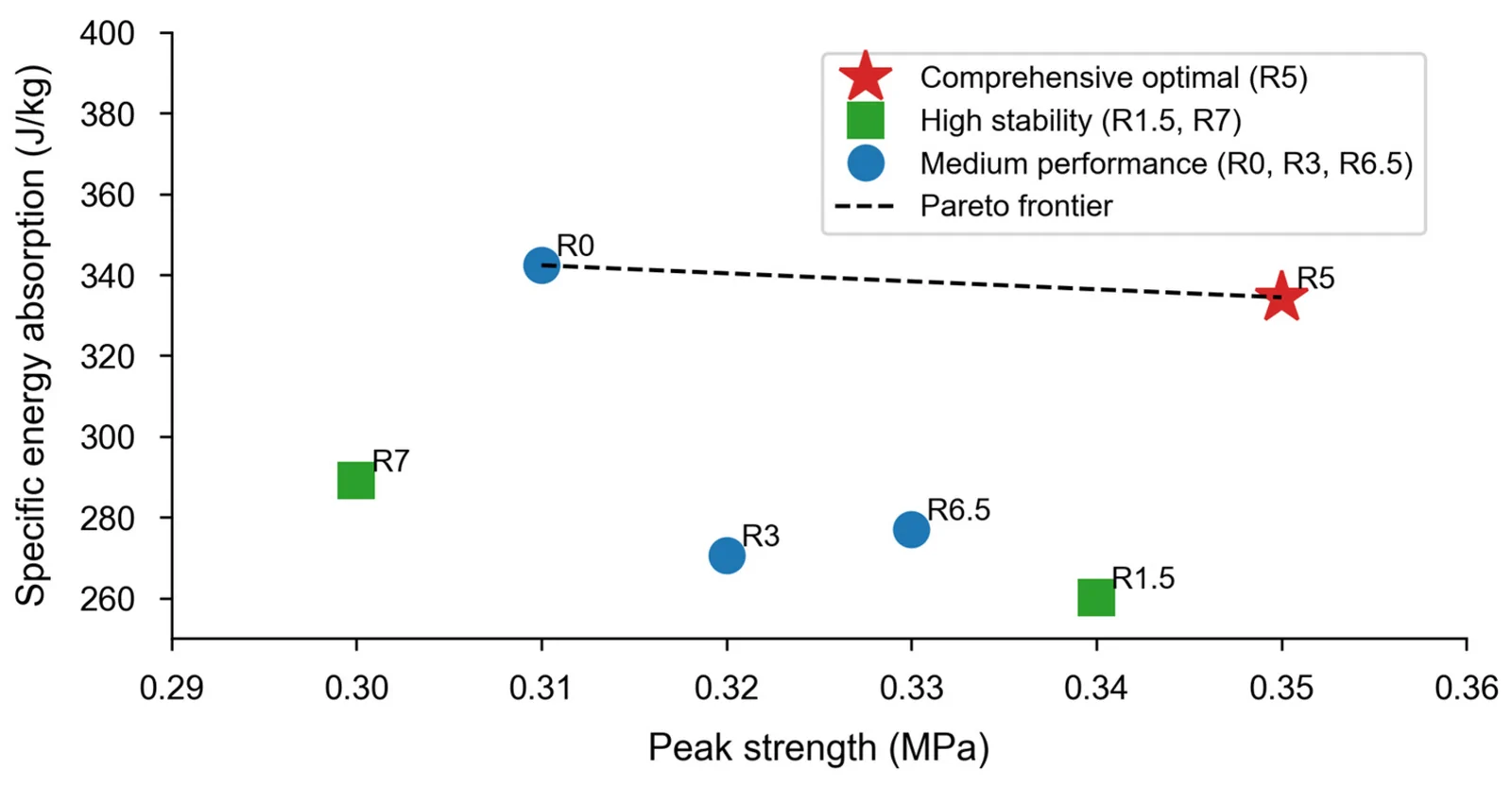
Pareto trade-off between peak strength and specific energy absorption (dashed line represents the Pareto optimal front). Red stars: R5 mm, best comprehensive performance; green squares: R1.5 and R7 mm, highest crushing smoothness (lowest CV); blue circles: R0, R3 and R6.5 mm, brittle or intermediate failure characteristics.

The Pareto trade-off relationship between peak strength and specific energy absorption is shown in Figure 9. The Pareto frontier is constructed by identifying non-dominated solutions: a configuration is defined as non-dominated if no other configuration achieves both higher peak strength and higher specific energy absorption simultaneously. Under the two-objective framework of strength and energy absorption, both R0 (highest SEA) and R5 (highest peak strength) lie on the Pareto frontier, representing two different performance trade-off directions.

As summarized in Table 5, the R5 mm specimens achieved the highest comprehensive score (0.764) under the weighted normalization of peak strength (0.4), specific energy absorption (0.4), and crushing stability (0.2), confirming that an intermediate fillet radius offers the best multi-objective trade-off among the six tested configurations. Detailed engineering application recommendations are given in Section 4.3.

For a more detailed characterization of the crushing stability, seven supplementary fluctuation-pattern indicators and a weighting-sensitivity analysis of the comprehensive ranking are provided in the Supplementary Materials (Tables S1 and S2).

## 4. Discussion

### 4.1. Dual Regulation Mechanism of Fillet Radius on Structural Failure Behavior

For PETG honeycombs, the dual regulatory mechanism of stress concentration mitigation and nodal cross-section weakening is inferred from macroscopic compression responses, failure modes and established porous mechanics theory, rather than directly verified by local stress field measurements. The interplay between these two mechanisms constitutes the intrinsic origin of the differing compressive failure behaviors among the specimen groups [11]. The geometric discontinuity at right-angle nodes creates stress concentration singularities. Notably, the elimination of stress concentration singularity refers to the removal of geometric discontinuity-induced infinite stress points, while the actual reduction amplitude of the stress concentration factor lacks direct quantitative measurement. Under compressive loading, the local stress gradient increases sharply, leading to rapid crack initiation and propagation, irregular brittle fracture, and multiple cliff-like drops in the load–displacement curve. This failure characteristic is consistent with previously reported behavior of honeycomb structures with right-angle nodes [33].

The R1.5 mm fillet is sufficient to eliminate the stress concentration singularity at the sharp right-angle node, and the degree of cross-section weakening is very slight, so the overall stress state at the node is effectively improved. For the R3 mm fillet, although the sharp-corner stress singularity is also eliminated and the corner stress gradient is further alleviated, the nodal load-bearing cross-section is more markedly weakened. The reduced cross-section leads to an elevated overall working stress level at the node, which superimposes with the residual stress gradient and presents brittle fracture behavior similar to stress concentration. These two superimposed adverse effects correspond to the frequent large-magnitude load mutations observed in the load–displacement curves in Figure 4 and further aggravate the suddenness of failure. In contrast, the R1.5 mm fillet can completely eliminate the stress concentration singularity and markedly improve crushing smoothness. However, the circular-arc curvature changes the load-bearing fulcrum of the ribs, reducing the critical buckling load. As a result, the specimens enter plastic deformation prematurely, shortening the effective energy absorption interval and making them suitable only for low-load smooth working conditions.

The intermediate R5 mm fillet reconstructs the nodal stress field, eliminating stress concentration and delaying crack propagation. The failure mode transforms into ordered layer-by-layer plastic crushing, corresponding to curves characterized by ordered progressive fluctuation without catastrophic load drops associated with brittle fracture shown in Figure 4. Meanwhile, the degree of cross-section weakening is moderate, retaining the structural load-bearing stiffness to the greatest extent [34]. The large-fillet R6.5 and R7 mm specimens also achieve nodal stress homogenization and exhibit a gentle crushing process. However, the excessive circular-arc transition substantially reduces the nodal cross-sectional area, causing a simultaneous decline in overall stiffness and peak strength. As a result, the upper bounds of load-bearing and energy absorption capacities are limited, making these configurations suitable only for low-load buffering conditions [30]. It should be noted that the above deduction of buckling, crack propagation and interlayer failure mechanisms is based on macroscopic load–displacement curves and failure morphologies, without direct in situ experimental observation. In situ characterization techniques such as high-speed imaging and DIC will be adopted in future research to directly verify the meso-scale failure process.

### 4.2. Core Mechanisms Underlying Differences in Structural Energy Absorption

According to the fundamental theory of energy absorption in porous media, the effective plastic deformation range and the stability of the plateau load jointly determine the energy absorption performance. As indicated by the quantitative data in Table 3, the R3 mm small-fillet specimens tend to induce early local brittle fracture, substantially shortening the effective energy absorption interval. The fluctuating load leads to considerable ineffective dissipation of plastic work, resulting in low values of total energy absorption, specific energy absorption, and energy absorption efficiency.

As also shown in Table 3, the R1.5 mm specimens exhibit small crushing fluctuations, but their relatively low average plateau stress leads to insufficient energy storage per unit deformation and the lowest energy absorption efficiency among all groups. The unfilleted R0 specimens, which have no cross-sectional loss at the nodes, achieve the highest total energy absorption; however, the right-angle-induced intermittent brittle collapse disrupts the continuity of deformation, so their numerical advantages cannot be translated into stable service performance. The R5 mm specimens show total and specific energy absorption only slightly lower than those of R0; owing to the orderly progressive crushing mode, ineffective energy dissipation is suppressed and the energy absorption efficiency is well balanced, yielding the best comprehensive energy absorption performance [32]. The R6.5 and R7 mm specimens exhibit smooth crushing processes, but the excessive cross-sectional weakening reduces the energy absorption density per unit deformation, making them incapable of meeting high-level buffering requirements.

In summary, an appropriate nodal fillet can synergistically balance load-bearing capacity, stability, and energy absorption performance. This concept is consistent with the balanced configuration optimization strategy for metamaterials [19,35].

### 4.3. Research Novelty and Engineering Application Value

Existing studies on porous metamaterials have mostly focused on irregular topologies such as star-shaped and chiral configurations [36,37], with metals and PLA as the primary base materials. Quantitative experimental systems for the fillet design of engineering-grade FDM-printed PETG auxetic honeycombs remain scarce [17], and no quantitative relationship among fillet size, failure behavior, and energy absorption has been established specifically for high-toughness PETG materials. In this study, the nodal fillet radius was taken as the sole variable in controlled compression tests, systematically revealing the dual coupling mechanism of stress optimization and cross-section weakening. The results enrich the experimental database for PETG polymer lattices and are distinct from existing studies based on metals or brittle PLA [38]. Using auxetic honeycombs as the carrier and a commercial PETG filament, multi-level gradient fillet compression tests were conducted to elucidate the dual regulation mechanism of the fillet and establish a quantitative correspondence between geometric parameters and macroscopic buffering performance.

Engineering selection recommendations:

1. For general lightweight crashworthiness and multi-load buffering conditions: based on the peak strength in Figure 6, the energy absorption indicators in Figure 7, and the Pareto front in Figure 9, the R5 mm configuration is recommended as the preferred choice owing to its best overall performance.
2. For low-impact, high-precision protective scenarios: based on the low load fluctuation coefficient shown in Figure 8, the R1.5 and R7 mm specimens are recommended.
3. Under the experimental conditions of this study (PETG material, specific honeycomb geometry, fixed FDM process and quasi-static loading), the R3 mm fillet exhibits relatively unfavorable comprehensive performance. This configuration yields the lowest comprehensive score of the six configurations (S = 0.231, rank 6 in Table 5), because it combines a below-average load-bearing and energy-absorption capacity (peak load 447.81 N, 4th of 6; W = 6.44 J, 5th of 6) with a high load fluctuation (CV = 44.15%, the third highest and numerically close to R0 at 44.17%); individually, it is not the weakest group in any single indicator. It also poses potential safety risks associated with brittle failure; it is therefore recommended to give priority to other configurations in engineering applications under similar conditions.

It should be noted that the stress concentration mitigation effect is inferred from macroscopic failure behaviors and load–displacement curves, without direct local stress characterization via finite element analysis or DIC measurement. Quantitative verification of the local stress field will be conducted in future research.

## 5. Conclusions

(1) The nodal fillet regulates the compressive performance of PETG honeycombs through the dual competing mechanisms of stress homogenization and load-bearing cross-section weakening, and the mechanical indicators vary nonlinearly with the fillet size.
(2) The R3 mm small-fillet configuration simultaneously exhibits superimposed stress elevation and cross-section weakening under the current experimental conditions, which readily induces brittle failure at the early stage of compression and results in the worst overall performance among the six tested configurations. The R1.5, R6.5, and R7 mm configurations can improve crushing smoothness, but the excessive circular-arc transition weakens the load-bearing and energy absorption limits, making them suitable only for specialized low-load conditions.
(3) The unfilleted R0 specimens achieve the highest total energy absorption, but the stress concentration at right-angle nodes induces disordered brittle collapse, and its reliability under dynamic impact conditions remains to be further verified. Among the six tested discrete fillet configurations, when the ratio of the nodal fillet radius to cell spacing is 35.7% (R5 mm), the most favorable balance between stress homogenization and cross-sectional load-bearing capacity is achieved under the adopted PETG material, fixed FDM process and quasi-static compression conditions, with a peak strength of 0.35 MPa and a specific energy absorption of 335.25 J/kg. The crushing process is dominated by ordered progressive buckling without catastrophic load drops associated with brittle fracture, and the comprehensive performance is optimal among the six tested discrete fillet configurations according to multi-indicator trade-off evaluation, making this configuration suitable for the vast majority of lightweight buffering scenarios. When the fillet ratio increases to 50% (R7 mm), the cross-section weakening effect outweighs the benefit of stress optimization, and the peak strength decreases to 0.30 MPa, which is even lower than that of the unfilleted baseline specimen. When the fillet ratio is below 21.4% (R3 mm), although the sharp-corner stress singularity is removed, the pronounced nodal cross-section weakening elevates the local working stress and triggers premature brittle fracture, resulting in comprehensively degraded performance. It should be noted that this optimal point is obtained based on six discrete gradient values; the precise optimal value in a continuous parameter range requires further experimental verification with denser parameter gradients.
(4) Nodal circular-arc filleting is a low-cost topological modification method. Reasonable matching of the fillet parameters can simultaneously balance structural load-bearing capacity, deformation stability, and energy absorption, thereby providing experimental support for similar thermoplastic 3D-printed energy-absorbing honeycombs.
(5) All performance laws and structural optimization conclusions obtained in this study are applicable to quasi-static compression conditions. This study was conducted only under quasi-static loading at room temperature with fixed specimen dimensions and a uniform FDM process, and did not investigate the coupled effects of printing layer thickness, cell aperture, and dynamic impact loading. The dynamic impact resistance of the filleted honeycomb structures needs to be further verified. Future work should include multi-variable coupled experiments and dynamic impact tests to improve the engineering design framework for filleted honeycomb structures [39].

## Figures and Tables

**Table 1:** Summary of key experimental parameters.

Category	Parameter	Value/Setting	Type
Geometric parameters	Overall specimen size	74 mm × 70 mm × 20 mm	Fixed
	Cell spacing	14 mm	Fixed
	Nominal rib width	1 mm	Fixed
	Node fillet radius	0, 1.5, 3, 5, 6.5, 7 mm	Variable
Material parameters	Filament material	PolyLite™ PETG (Polymaker)	Fixed
	Filament diameter	1.75 ± 0.02 mm	Fixed
	Material density	1.27–1.28 g/cm^3^	Fixed
FDM process parameters	Printer model	Ender-3 S1 Pro	Fixed
	Nozzle diameter	0.4 mm (brass)	Fixed
	Nozzle temperature	230 °C	Fixed
	Bed temperature	60 °C	Fixed
	Layer height	0.20 mm	Fixed
	Printing speed	50 mm/s	Fixed
	Infill density	60%	Fixed
Compression test parameters	Loading mode	Displacement control	Fixed
	Loading rate	5 mm/min	Fixed
	Maximum displacement	≈40 mm (recorded to 40.8 mm)	Fixed
	Test standard	GB/T 31930-2015/ISO 13314:2011	Fixed

**Table 2:** Peak load and peak strength of different specimens. The values in parentheses denote the fillet-radius-to-cell-spacing ratio. ^a^ Relative to the unfilleted R0 control group. All values are presented as mean ± standard deviation (SD), calculated from three replicate specimens per group.

Specimen	R0	R1.5 (10.7%)	R3 (21.4%)	R5 (35.7%)	R6.5 (46.4%)	R7 (50.0%)
Peak load (N)	429.98 ± 12.96	469.12 ± 33.12	447.81 ± 7.54	493.42 ± 7.53	458.85 ± 13.18	426.05 ± 23.59
Peak strength (MPa)	0.31 ± 0.01	0.34 ± 0.02	0.32 ± 0.01	0.35 ± 0.01	0.33 ± 0.01	0.30 ± 0.02
Relative peak strength (%) ^a^	100	109.10	104.15	114.75	106.71	99.08
Mass (g)	24.04 ± 0.26	23.96 ± 0.59	23.78 ± 0.31	23.72 ± 0.78	23.51 ± 0.94	23.39 ± 0.80
Relative density (%)	18.21 ± 0.20	18.15 ± 0.45	18.01 ± 0.23	17.97 ± 0.59	17.81 ± 0.71	17.72 ± 0.61

**Table 3:** Energy absorption performance indicators of different specimens. All values are presented as mean ± standard deviation (SD).

Specimen	R0	R1.5 (10.7%)	R3 (21.4%)	R5 (35.7%)	R6.5 (46.4%)	R7 (50.0%)
Total energy absorption, W (J)	8.24 ± 0.09	6.24 ± 0.15	6.44 ± 0.28	7.95 ± 0.26	6.57 ± 0.26	6.83 ± 0.23
Specific energy absorption, SEA (J/kg)	342.62 ± 3.86	260.64 ± 6.44	270.88 ± 11.78	335.25 ± 11.02	279.45 ± 11.09	292.05 ± 9.92
Energy absorption efficiency (%)	46.97 ± 1.21	32.75 ± 2.65	35.28 ± 2.12	39.51 ± 1.38	35.12 ± 2.07	39.35 ± 1.64

**Table 4:** Comparison of crushing stability of samples with different fillet radii. All values are presented as mean ± standard deviation (SD). CV was calculated over the fixed displacement window of 2–30 mm (see Section 2.4); it reflects the overall dispersion of the plateau load and is used as an auxiliary, not primary, stability indicator.

Specimen	R0	R1.5 (10.7%)	R3 (21.4%)	R5 (35.7%)	R6.5 (46.4%)	R7 (50.0%)
Load fluctuation coefficient (%)	44.17 ± 1.24	28.42 ± 1.53	44.15 ± 6.04	49.69 ± 4.01	42.50 ± 1.17	38.86 ± 0.64

**Table 5:** Normalized performance scores and comprehensive ranking of the six fillet configurations.

Specimen	R0	R1.5 (10.7%)	R3 (21.4%)	R5 (35.7%)	R6.5 (46.4%)	R7 (50.0%)
Peak strength	0.058	0.639	0.323	1.000	0.487	0.000
Specific energy absorption	1.000	0.000	0.125	0.910	0.229	0.383
Crushing stability	0.259	1.000	0.260	0.000	0.338	0.509
Comprehensive score	0.475	0.456	0.231	0.764	0.354	0.255
Rank	2	3	6	1	4	5

## Data Availability

The original contributions presented in this study are included in the article/Supplementary Materials. Further inquiries can be directed to the corresponding author.

## Supplementary Materials

The following supporting information can be downloaded at: https://www.mdpi.com/article/10.3390/polym18182226/s1, Table S1: Supplementary fluctuation-pattern indicators (number of peaks Np, peak density PD, mean prominence Prom, mean inter-peak drop Mdrop, normalised mean inter-peak drop NMID, coefficient of variation of inter-peak drops CVdrops and burst ratio BR) for the six fillet configurations; Table S2: Sensitivity of the comprehensive score and ranking to the choice of weighting coefficients (171 weighting combinations); Table S3: Mass-normalised strength comparison (peak load normalised by specimen mass, Fmax/m, versus nominal strength).
