# Supplementary material for: Effects of Node Fillet Radius on Compressive Properties and Energy Absorption of FDM-Fabricated PETG Auxetic Honeycombs
Source: Polymers (Basel). 2026 Sep 12;18(18):2226. doi: 10.3390/polym18182226 (PMC13610998; doi:10.3390/polym18182226)
Supplement: Supplementary file 1 [file polymers-18-02226-s001.zip › polymers-4535162_supplementary.pdf]

Supplementary Materials

Effects of Node Fillet Radius on Compressive Properties and Energy Absorption of FDM-Fabricated PETG Auxetic Honeycombs

Xin Kuang, Zhangyong Hu, Ruiyong Duan and Guangchao Zhan *

School of Electromechanical and Intelligent Manufacturing, Huanggang Normal University, Huanggang 438000, China
* Correspondence: guangchaozhan@hgnu.edu.cn

S1. Fluctuation-Pattern Indicators

To complement the load fluctuation coefficient (CV), which only captures the overall dispersion of the plateau load, seven supplementary indicators were derived from the smoothed force–displacement curves to distinguish periodic from sudden-drop fluctuation patterns: (i) Number of peaks (N_p) — the total number of prominent peaks detected by the peak-detection procedure; (ii) Peak density (PD, mm^{-1}) — the number of prominent peaks per unit displacement, obtained by Savitzky–Golay smoothing (window ≈ 0.2 mm, polynomial order 3) followed by peak detection (prominence ≥ 30 N, minimum spacing 1.0 mm); (iii) Mean prominence (Prom, N) — the average prominence of all detected peaks, i.e. the height difference between each peak and the lowest point on the adjacent higher peak; (iv) Mean inter-peak drop (M¯_drop, N) — the arithmetic mean of the load drops between consecutive peaks (peak value minus the intervening valley value); (v) Normalized mean inter-peak drop (NMID) — the mean magnitude of the load drop between two consecutive peaks, divided by the mean load over the full 0–40.8 mm record; (vi) Coefficient of variation of inter-peak drops (CV_drops) — small values indicate uniform/periodic drops, large values indicate irregular drops; (vii) Burst ratio (BR) — the ratio of the maximum to the mean inter-peak drop, with values close to 1 denoting periodic behavior and values substantially greater than 1 indicating the presence of a dominant sudden drop. A specimen is classified as periodic when CV_drops < 30% and BR < 1.5; as sudden-drop when BR > 1.8; as irregular when CV_drops > 45%; otherwise as mixed.

Table S1. Supplementary fluctuation-pattern indicators for the six fillet configurations (values are means of three replicates). a CV is calculated over the plateau window of 2–30 mm, i.e. using exactly the same definition as in Table 4, so that the two tables are directly comparable; it differs from a coefficient computed over the entire 0–40.8 mm record, which would additionally contain the initial elastic rise and the peak load and is therefore not a measure of plateau undulation. The seven supplementary indicators (N_p, PD, Prom, M¯_drop, NMID, CV_drops, BR) are derived from the Savitzky–Golay-smoothed full record by the peak-detection procedure given in Section S1, and their absolute magnitudes are mildly sensitive to the smoothing parameters, whereas the pattern classification is robust. For completeness, the coefficients computed over the entire 0–40.8 mm record are 43.23, 50.73, 43.58, 46.97, 42.33 and 41.79% for R0–R7; because this interval additionally contains the initial elastic rise and the peak load, the full-record coefficient is governed by the initial peak-to-plateau ratio (e.g. R1.5: peak 469 N against a mean load of ≈152 N over the full 0–40.8 mm record) rather than by plateau undulation, and must not be interpreted as a measure of crushing stability.

Specimen	R0	R1.5	R3	R5	R6.5	R7
**CV (%) a**	44.17	28.42	44.15	49.69	42.50	38.86
**N_p**	5.3	4.7	5.3	4.0	4.0	4.0
**PD (mm^{-1})**	0.1541	0.1382	0.1467	0.1363	0.1294	0.1247
**Prom (N)**	179.84	107.28	174.33	249.16	224.79	193.32
**$\bar{M}$_drop (N)**	190.04	89.38	203.45	271.69	239.82	185.99
**NMID**	0.941	0.585	1.289	1.393	1.491	1.112
**CV_drops (%)**	36.59	27.41	44.44	22.55	36.16	51.44
**BR**	1.56	1.30	1.70	1.28	1.36	1.72
**Pattern**	mixed	periodic	mixed	periodic	mixed	irregular

Despite the comparable CV values of the R0 (44.17%), R3 (44.15%) and R5 (49.69%) specimens, the supplementary indicators reveal fundamentally different fluctuation patterns. The R5 specimens exhibit the lowest CV_drops (22.55%) and the smallest burst ratio (1.28) among all groups, indicating that their relatively high CV originates from a periodic, layer-by-layer plastic collapse rather than from sporadic catastrophic drops. In contrast, the R7 specimens show the highest CV_drops (51.44%), consistent with their irregular and non-reproducible failure mode. These quantitative findings corroborate the qualitative observation that the R5 configuration achieves an ordered progressive crushing behavior with stable, repeatable load oscillations.

S2. Weight Sensitivity of the Comprehensive Ranking

To assess the robustness of the comprehensive ranking against the choice of weighting coefficients, the three-dimensional weighting simplex ($w_1 + w_2 + w_3 = 1$, where w_1, w_2, w_3 are the weights assigned to peak strength, specific energy absorption and crushing stability, respectively) was scanned with a uniform step of $\Delta w = 0.05$ (171 valid combinations in total). For each weighting combination, the comprehensive score of every specimen was recomputed using the normalized indicators in Table 5 and the specimens were re-ranked.

Table S2. Sensitivity of the comprehensive score and ranking to the choice of weighting coefficients. S = comprehensive score; R = rank. Each cell shows S (R). Scores are rounded to three decimals; where two scores coincide at this precision they are reported as a tie and receive the same rank (2=), and ranking follows the standard competition convention (the next distinct score receives rank 4). In the stability-leaning scenario the R5 and R7 scores are 0.3820 and 0.3821, a difference of 1×10^{-4} that is far below the resolution of the underlying measurements; they are therefore treated as tied for second place.

Weight scenario ($w_1/w_2/w_3$)	R0	R1.5	R3	R5	R6.5	R7
Original (0.40/0.40/0.20)	0.475 (2)	0.456 (3)	0.231 (6)	0.764 (1)	0.354 (4)	0.255 (5)
Peak-leaning (0.60/0.20/0.20)	0.287 (4)	0.584 (2)	0.271 (5)	0.782 (1)	0.406 (3)	0.178 (6)
Energy-leaning (0.30/0.50/0.20)	0.569 (2)	0.392 (3)	0.211 (6)	0.755 (1)	0.328 (4)	0.293 (5)
Stability-leaning (0.20/0.20/0.60)	0.367 (4)	0.728 (1)	0.246 (6)	0.382 (2=)	0.346 (5)	0.382 (2=)

The R5 mm configuration ranks first in 52.0% of all valid weighting combinations (89 out of 171) and remains within the top three in 85.4% of scenarios. R5 fails to rank first in the remaining 82 combinations. R0 prevails in 19 of them, all of which combine only a marginal weight on peak strength ($w_1 \leq 0.15$) with a dominant weight on energy absorption ($w_2 \geq 0.45$), a regime governed by the highest SEA of R0 (342.62 J kg^{-1}). The R1.5 mm specimens prevail in the other 63 combinations, all of which satisfy $w_3 \geq 0.30$, i.e. R1.5 requires at least a moderate weight on crushing stability, under which its lower and

smoother plateau load is rewarded; 44 of these 63 have $w_3 \geq 0.50$. Analytically, R5 ranks first if and only if $0.942 \cdot w_1 > 0.090 \cdot w_2 + 0.259 \cdot w_3$ and $0.549 \cdot w_1 + 1.910 \cdot w_3 < 0.910$. Because practical energy-absorbing structures must balance load-bearing capacity and energy absorption rather than stability alone, the original (0.40, 0.40, 0.20) weighting scheme — which assigns equal emphasis to peak strength and energy absorption while preserving a non-negligible contribution from stability — is both physically justified and statistically robust.

S3. Mass-Normalized Strength Comparison

Because the effective load-bearing cross-sectional area of the ribs cannot be determined without arbitrary geometric assumptions, the corresponding normalization was not performed directly. As the closest assumption-free proxy, the peak load was normalized by the measured specimen mass (Fmax/m); the results are given in Table S3. The mass-normalized ranking is R5 > R1.5 > R6.5 > R3 > R7 > R0, which is not identical to the ranking by nominal strength (R5 > R1.5 > R6.5 > R3 > R0 > R7): the two lowest-ranked configurations, R0 and R7, interchange. Both normalizations place R5 first, so the core conclusion that R5 is the optimal configuration is unaffected. Because specimen mass is only a proxy for, and not identical to, the effective load-bearing area, this check is indicative rather than a substitute for a true effective-area normalization.

Table S3. Mass-normalized strength comparison: peak load normalized by specimen mass (Fmax/m) versus nominal strength σ = Fmax/A0. Values are group means of three replicates.

Group	R0	R1.5	R3	R5	R6.5	R7
**Peak load Fmax (N)**	429.98	469.12	447.81	493.42	458.85	426.05
**Group mean mass (g)**	24.04	23.96	23.78	23.72	23.51	23.39
**Nominal σ (MPa)**	0.3071	0.3351	0.3199	0.3524	0.3278	0.3043
**Mass-normalized strength F/mass (N/g)**	17.89	19.58	18.83	20.80	19.52	18.21
**Rank by σ**	5	2	4	1	3	6
**Rank by F/mass**	6	2	4	1	3	5

Note: R0 and R7 interchange between the two normalizations (nominal σ: R0 > R7; Fmax/m: R7 > R0), but both remain below R5, so this adjacent-pair reordering does not affect the optimum. R5 ranks first under both normalizations.
